# Heat shock protein 27 phosphorylation state is associated with cancer progression

**DOI:** 10.3389/fgene.2014.00346

**Published:** 2014-10-06

**Authors:** Maria Katsogiannou, Claudia Andrieu, Palma Rocchi

**Affiliations:** ^1^Institut National de la Santé et de la Recherche Médicale, Unités Mixtes de Recherche 1068, Centre de Recherche en Cancérologie de MarseilleMarseille, France; ^2^Institut Paoli-CalmettesMarseille, France; ^3^Centre de Recherche en Cancérologie de Marseille, Institut National de la Santé et de la Recherche Médicale Unités Mixtes de Recherche 1068, Aix-Marseille UniversitéMarseille, France; ^4^Centre National de la Recherche Scientifique, Unités Mixtes de Recherche 7258, Centre de Recherche en Cancérologie de MarseilleMarseille, France

**Keywords:** Hsp27, phosphorylation, stress-induced, cancer, apoptosis resistance

## Abstract

Understanding the mechanisms that control stress-induced survival is critical to explain how tumors frequently resist to treatment and to improve current anti-cancer therapies. Cancer cells are able to cope with stress and escape drug toxicity by regulating heat shock proteins (Hsps) expression and function. Hsp27 (HSPB1), a member of the small Hsp family, represents one of the key players of many signaling pathways contributing to tumorigenicity, treatment resistance, and apoptosis inhibition. Hsp27 is overexpressed in many types of cancer and its functions are regulated by post-translational modifications, such as phosphorylation. Protein phosphorylation is the most widespread signaling mechanism in eukaryotic cells, and it is involved in all fundamental cellular processes. Aberrant phosphorylation of Hsp27 has been associated with cancer but the molecular mechanisms by which it is implicated in cancer development and progression remain undefined. This mini-review focuses on the role of phosphorylation in Hsp27 functions in cancer cells and its potential usefulness as therapeutic target in cancer.

## INTRODUCTION

Protein phosphorylation is the most widespread post-translational modification in eukaryotic cells, and it is involved in all fundamental cellular processes. Reversible phosphorylation based signaling networks are crucial to the cell’s capacity to quickly respond to external and internal stimuli. An estimated 30% of cellular proteins are phosphorylated in an estimated total of 1000s of distinct phosphorylation sites (Cohen, 2000). This post-translational modification plays a crucial role in the cellular functions of proteins such as heat shock proteins (Hsps), particularly Hsp27 (HSPB1; Kostenko and Moens, 2009). Hsp27 is an ATP-independent molecular chaperone with well-described tumorigenic and metastatic roles, characterized by its dynamic phosphorylation leading to heterogeneous oligomerization under different conditions such as stress (Jakob et al., 1993; Martin et al., 1999; Garrido, 2002; Gusev et al., 2002; Kato et al., 2002; Koteiche and McHaourab, 2003; Webster, 2003; Taylor and Benjamin, 2005; Acunzo et al., 2012). Unphosphorylated Hsp27 is able to form multimers than can reach 800 kDa (Lentze and Narberhaus, 2004) while phosphorylation results in conformational changes leading to significantly decreased oligomeric size, complex dissociation, and subsequent loss of chaperone activity (Rogalla et al., 1999; Hayes et al., 2009). This supports the idea that Hsp27’s reversible structural organization acts as a sensor allowing cells to adapt and eventually overcome lethal conditions by interacting with appropriate protein partners (Arrigo and Gibert, 2012).

It has been well documented that aberrations in protein phosphorylation are closely linked to major diseases such as cancer, diabetes, and rheumatoid arthritis (Radivojac et al., 2008; Watanabe and Osada, 2012; Hao et al., 2013; Nie et al., 2013; Streit et al., 2013). Moreover, Hsp27 overexpression contributes to the malignant progression of cancer cells including increased tumorigenicity, treatment resistance, and apoptosis inhibition (Hsu et al., 2011; Acunzo et al., 2012; Stope et al., 2012). While the aberrant expression of Hsp27 in human cancer have been and is still intensively studied and documented, its phosphorylation state in cancer cells compared to healthy cells are only starting to be examined (Arrigo et al., 2007; Calderwood and Ciocca, 2008; Arrigo and Gibert, 2012). Hsp27 is not the only chaperone whose functions in cancer cells are coordinated by phosphorylation regulation. A recent study identified C-terminal phosphorylation as a key mechanism for the dynamic regulation of Hsp90 and Hsp70 chaperone activity, and binding to co-chaperones to either fold or degrade client proteins (Muller et al., 2013). These co-chaperones are also regulated in a way that favors pro-folding environment in replicating tumor cells and degradation phenotype in non-proliferating cells (Muller et al., 2013). This mini-review briefly summarizes the regulation of Hsp27 by phosphorylation and its functional implications and focuses on the reports describing aberrant Hsp27 phosphorylation linked to cancer. The potential therapeutic strategies aiming at Hsp27 phosphorylation will also be discussed as future perspectives.

## Hsp27 PHOSPHORYLATION AND ASSOCIATED FUNCTIONS IN NORMAL CELLS: AN UP-TO-DATE OVERVIEW

**FIGURE 1:**
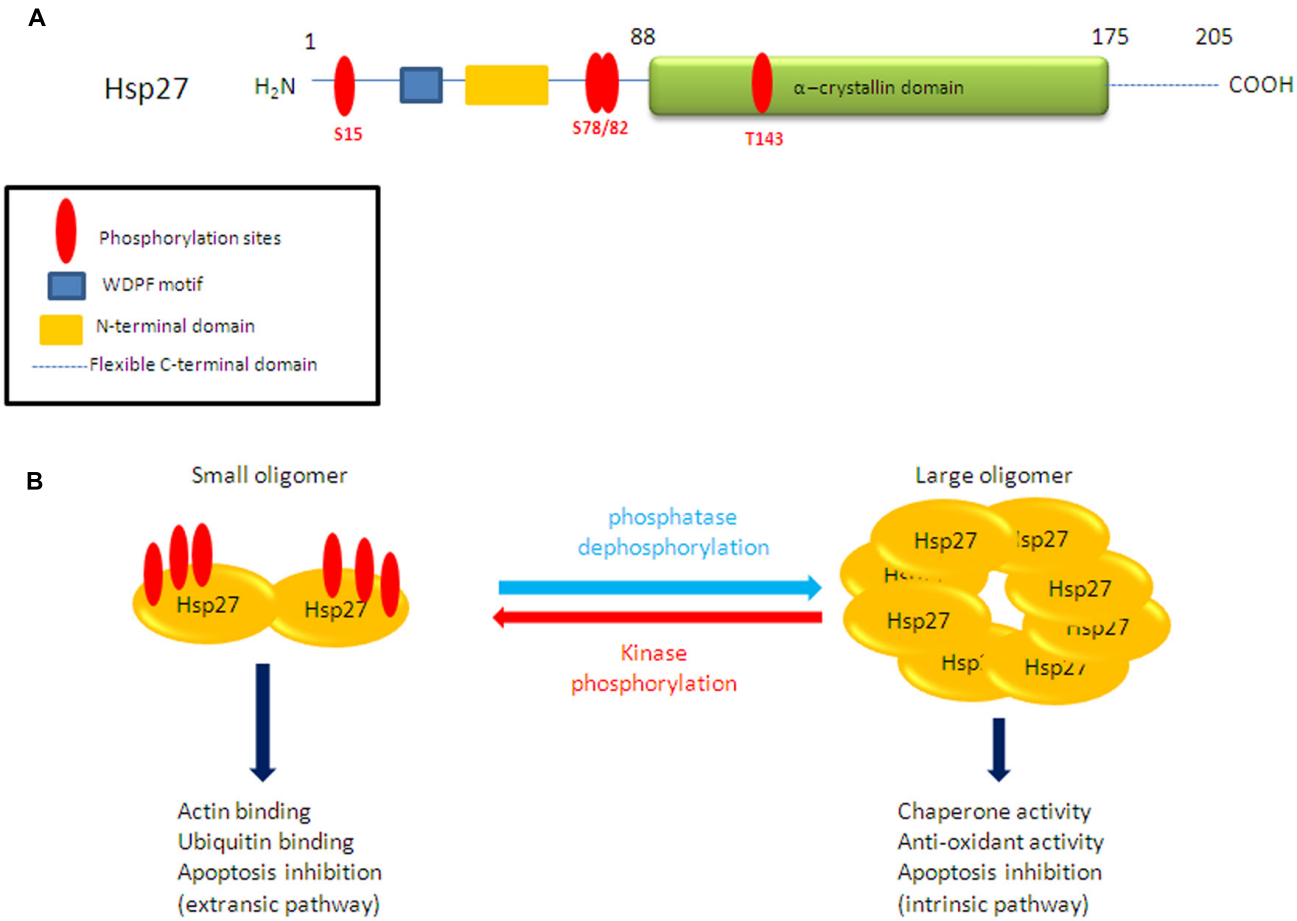
**(A)** Schematic representation Hsp27 structure and putative phosphorylation sites. **(B)** Structural organization of Hsp27 upon reversible phosphorylation.

Mapping the phosphorylation sites of Hsp27 showed the involvement of Serine (Ser)-15, Ser-82, Ser-78, and Threonine (Thr-143) residues (**Figure 1A**). The contribution of single phosphorylation of Hsp27 at either of these sites, in biological processes has not yet been addressed. However, previous studies have shown that Hsp27 oligomerization is regulated by Ser-78 and/or Ser-82 phosphorylation while Ser-15 seems to induce small effect on oligomerization (Lambert et al., 1999; Gusev et al., 2002). Hsp27 phosphorylation/dephosphorylation equilibrium (**Figure 1B**) has been shown to be regulated by signals activating protein kinases and phosphatases. Numerous *in vitro* and *in vivo* studies in different cell types have described the roles of MAPK-activated protein kinase–2,–3,–5 (MK2, MK3, MK5), protein kinase (PK) A, B, C, and D in Hsp27 phosphorylation [for review (Kostenko and Moens, 2009)]. The choice of the kinase seems to depend on the cell type therefore kinase expression levels and the signaling pathway activated. Even though numerous kinases have been described to interact with and/or phosphorylate directly or indirectly Hsp27, controversy exists on the subject and the major kinases have been shown to be MK2, MK5, and PKD (Doppler et al., 2005). When induced upon stress, Hsp27 phosphorylation can be detected within a few minutes (Landry et al., 1992) and in a reversible manner which is controlled by phosphatases. Several studies have revealed the involvement of protein phosphatase 2A (PP2A; Cairns et al., 1994; Tar et al., 2006) but the involvement of other protein phosphatases is not excluded.

In addition to controlling Hsp27 structural organization and oligomerization, phosphorylation seems to be a key mechanism which favors recognition of specific client proteins associating Hsp27 with specific functions (Arrigo and Gibert, 2012, 2013). Several functions are associated with Hsp27 phosphorylation in normal cells (**Figure 1B**). It has been well described that phosphorylated Hsp27 regulates actin filaments dynamics, in cytoskeleton organization during processes such as cell migration or cell stress (Lavoie et al., 1993; Clarke and Mearow, 2013). Various studies have also shown that Hsp27 overexpression and/or phosphorylation regulates cell cycle and therefore cell proliferation but this appears to be cell-specific. It has been demonstrated that phosphoHsp27 inhibited the MEK/ERK signaling pathway by a mechanism involving both c-Raf activity attenuation and stimulation of MAPK phosphatase-1 (MKP1) through p38 MAPK leading to significant reduction of cyclin D1 levels and subsequent cell cycle arrest (Matsushima-Nishiwaki et al., 2008). Moreover, Hsp27 is known to interact with p53, regulating its transcriptional activity (Venkatakrishnan et al., 2008), therefore having an effect in cell cycle regulation. Last but not least, phosphoHsp27 can prevent apoptosis by protecting cells against heat shock, apoptosis effectors, oxidative stress, and ischemia. Hsp27 can also inactivate Bax and block the release of Smac and cytochrome C (Garrido et al., 2006; Arrigo, 2007; Acunzo et al., 2012). It is important to note at this point that in cells treated by apoptotic effectors that act on different pathways, Hsp27 has diverse localizations, oligomeric sizes, and phosphorylation states leading to negative regulation of apoptosis (Paul et al., 2010). More precisely, the two apoptotic effectors, etoposide, and Fas antibody, have the tendency to increase Hsp27 native sizes reflecting medium sized and large oligomers accumulation, while staurosporine and cytochalasin D induced Hsp27 in small oligomers (Paul et al., 2010). Hsp27 acts by regulating partner proteins involved in cell death pathways (Havasi et al., 2008; Acunzo et al., 2012; Sanchez-Nino et al., 2012).

## THE RATIONAL OF TARGETING Hsp27 ABERRANT PHOSPHORYLATION IN CANCER

Aberrant expression of Hsp27 in cancer cells has been intensively investigated and is known to be associated with aggressive tumor phenotype, increased therapy resistance, and poor prognosis for the patient. Targeting Hsp27 overexpression in different types of cancers has been shown promising (Rocchi et al., 2006; Cayado-Gutierrez et al., 2013; Lamoureux et al., 2014) but currently no clinical trial has passed phase III (Agensys, 2014). However, less focus has been given to the phosphorylation state of Hsp27 in cancer cells compared to healthy ones. Interestingly, a few recent studies demonstrated that phosphorylation levels of Hsp27 increased in advanced tumors and were correlated to treatment resistance (Taba et al., 2010; Wang et al., 2010; Sakai et al., 2012; Xu et al., 2014). A proteomics study identified phosphoHsp27 as part of the cancer-related phosphoprotein signature of prostate cancer (Chen et al., 2011). In the described study, phosphorylation of Hsp27 occurs upon androgen receptor (AR) activation by ligands, leading to Hsp90 displacement from the AR-complex and translocation of AR to the nucleus. Inhibition of Hsp27 phosphorylation shifted the association of AR with Hsp90 to the E3 ubiquitin ligase MDM2, increased AR degradation, decreased AR transcriptional activity and increased prostate cancer cell apoptotic rates (Chen et al., 2011). In pancreatic and prostate cancer cells, cytoprotection induced by Hsp27 is due, at least in part, to its interaction with eIF4E (eukaryotic translational initiation factor 4E) that increased when Hsp27 is phosphorylated. Hsp27 interaction protects eIF4E from its ubiquitin-proteasome-dependent degradation process, leading to apoptosis resistance induced by castration and chemotherapy (Andrieu et al., 2010; Baylot et al., 2011). A similar mechanism was previously described involving cooperative interactions between ligand-activated AR and Hsp27 phospho-activation that enhance AR stability, shuttling, and transcriptional activity, thereby increasing prostate cancer cell survival (Zoubeidi et al., 2007). Moreover, Hsp27 phosphorylation has been shown to regulate epithelial–mesenchymal transition process and NF-B activity contributing to the maintenance of breast cancer stem cells (Wei et al., 2011). A comparative phosphoproteomic studies of HER-2/neu positive and -negative breast tumors revealed that Hsp27, one of the identified phosphoproteins, was highly phosphorylated on Ser78 in HER-2/neu positive tumors (Zhang et al., 2007). In MCF-7 cells, phosphoHsp27 plays different roles in regulating p53 pathway and cell survival (Xu et al., 2013). In ovarian and prostate cancers, p38 MAPK-MK2 dependent phosphorylation of Hsp27 was shown to be involved in remodeling of actin filaments required for pro-invasive and pro-metastatic activities (Gurgis et al., 2014; Pavan et al., 2014). Interestingly, the authors propose targeting the MK2-Hsp27 axis in cancer cells as a strategy to reduce migration and metastasis in cancer cells. In a recent report, it was demonstrated that Hsp27 phosphorylation in liver cancer cells was associated with Hsp27 subcellular localization in the nucleus where it could perform specific functions such as mRNA processing (Bryantsev et al., 2007; Guo et al., 2012). Finally, Taba et al. (Taba et al., 2010) recently showed that phosphorylated Hsp27 played an important role in resistant to Gemcitabine in pancreatic cancer cells and propose phosphoHsp27 as a possible biomarker for predicting response of pancreatic cancer patients to Gemcitabine treatment.

In addition to targeting Hsp27 expression in cancer cells, it therefore appears of particular interest, to block the functions of phosphoHsp27. This approach may lead to new anti-cancer drug discovery. Less specific targeting strategies of the p38-MAPK signaling cascade have shown significant therapeutic potential in the treatment of endocrine resistant breast cancer through inhibition of downstream targets Hsp27 and MAPK (Antoon et al., 2012). Gibert et al. (Gibert et al., 2011) developed peptide aptamers that specifically bind Hsp27, interfere with its structural organization (dimerization and oligomerization) and impair its anti-apoptotic and cytoprotective functions. We believe that interfering with specific phosphoHsp27-partner protein interactions in cancer cells may represent a promising therapeutic strategy with little or no side effects in normal cells. Targeting phosphoHsp27 in cancer cells constitutes a nascent field research that deserves more exploration in the future.

## CONCLUSION AND FUTURE DIRECTIONS

The future challenge lies in a deeper understanding of Hsp27 phosphorylation state in cancer cells in order to develop and/or improve therapies, specific to cancer cells. The role of Hsp27 phosphorylation in cancer progression has only started to be explored and the few studies published to date that are described in this mini-review suggest that phosphoHsp27 suppresses apoptosis, enhances invasion, and survival of cancer cells. Interestingly, some elements suggest that phosphoHsp27 could present modified subcellular localization which may account for specific roles in cancer cells. We believe that apart from a thorough understanding of Hsp27 phosphorylation state in cancer cells, subcellular localization, and protein partner interactions of phosphoHsp27 are aspects that require further exploration as they will certainly reveal new cancer-specific functions for Hsp27. Interfering with Hsp27’s functions should be directed toward cancer cells considering the diversity of functions that Hsp27 exerts in normal cells. Several inhibitors against some Hsp27 kinases have been developed (Anderson et al., 2007; Schlapbach et al., 2008; Lopes et al., 2009) but clinical trials in patients with aberrant Hsp27 phosphorylation are lacking. Proteomics approaches are increasingly used to identify stress-induced chaperone phosphorylation in different pathophysiological conditions and may constitute useful tools for selecting patients who may respond to newly developed therapies.

## Conflict of Interest Statement

The authors declare that the research was conducted in the absence of any commercial or financial relationships that could be construed as a potential conflict of interest.
