# Correction: Heat shock protein 27 phosphorylation state is associated with cancer progression

**DOI:** 10.3389/fgene.2025.1703072

**Published:** 2025-10-23

**Authors:** Maria Katsogiannou, Claudia Andrieu, Palma Rocchi

**Affiliations:** ^1^ Institut National de la Santé et de la Recherche Médicale, Unités Mixtes de Recherche 1068, Centre de Recherche en Cancérologie de Marseille, Marseille, France; ^2^ Institut Paoli-Calmettes, Marseille, France; ^3^ Centre de Recherche en Cancérologie de Marseille, Institut National de la Santé et de la Recherche Médicale Unités Mixtes de Recherche 1068, Aix-Marseille Université, Marseille, France; ^4^ Centre National de la Recherche Scientifique, Unités Mixtes de Recherche 7258, Centre de Recherche en Cancérologie de Marseille, Marseille, France

**Keywords:** Hsp27, phosphorylation, stress-induced, cancer, apoptosis resistance

The conflict of interest statement was erroneously given as “The authors declare that the research was conducted in the absence of any commercial or financial relationships that could be construed as a potential conflict of interest.” The correct conflict of interest statement is “The University of British Columbia has submitted patent applications on Apatorsen, an antisense inhibitor of Hsp27, listing PR as an inventor. This IP has been licensed to OncoGenex Technologies, a Vancouver-based biotechnology company. The remaining authors declare that the research was conducted in the absence of any commercial or financial relationships that could be construed as a potential conflict of interest.”

The original article has been updated.

